# P-1782. Evolutionary Dynamics of Respiratory Syncytial Virus in Pre-pandemic, Pandemic, and Post-pandemic Periods in Houston, Texas, USA

**DOI:** 10.1093/ofid/ofaf695.1951

**Published:** 2026-01-11

**Authors:** Vasanthi Avadhanula, Daniel Agustinho, Leila C Sahni, Anil Sarathu, David M Henke, Harshavardhan Doddapaneni, Donna Muzny, Ginger A Metcalf, Sara J Javornik Cregeen, Natalie J Thornburg, Heidi L Moline, Ayzsa Tannis, Fritz Sedlazeck, Pedro A Piedra

**Affiliations:** Baylor College of Medicine, Houston, TX; Baylor College of Medicine, Houston, TX; Baylor College of Medicine and Texas Children's Hospital, Houston, Texas; Baylor College of Medicine, Houston, TX; Baylor College of Medicine, Houston, TX; Baylor College Medicine, Houston, Texas; Baylor College of Medicine, Human Genome Sequencing Center, HOUSTON, Texas; Baylor College of Medicine, Houston, TX; Alkek Center for Metagenomics and Microbiome Research, Dept. of Molecular Virology and Microbiology, Baylor College of Medicine, Houston, Texas; Centers for Disease Control and Prevention, Atlanta, Georgia; US-CDC, Atlanta, Georgia; Centers for Disease Control and Prevention, Atlanta, Georgia; Human Genome Sequencing Center, Baylor College of Medicine, Houston, TX, USA, Houston, Texas; Baylor College of Medicine, Houston, TX

## Abstract

**Background:**

Respiratory Syncytial Virus (RSV) remains a significant cause of respiratory illness in infants and older adults worldwide. The COVID-19 pandemic disrupted typical RSV fall/winter seasonality, leading to unusual patterns of viral circulation and resurgence. We investigated the evolutionary dynamics of RSV in Houston, Texas, over a nine-year period (2015–2024), encompassing pre-pandemic, pandemic, and post-pandemic phases.

**Methods:**

We sequenced 606 RSV/A and 570 RSV/B nasal swab or throat/nasal swabs samples from children outpatient clinic or in hospital with acute respiratory infections between November 1^st^, 2015, and Feb 1^st^, 2024, from the Houston site of the New Vaccine Surveillance Network. We assessed genetic diversity, lineage dynamics, and selective pressures, by phylogenetic analysis, variant calling, dN/dS ratio calculations and Shannon entropy.

**Results:**

Phylogenetic analysis revealed distinct lineage dynamics, with RSV/A showing persistence of certain pre-pandemic lineages (e.g., A.D.1) and the emergence of new ones (e.g., A.D.3) during pandemic and post-pandemic periods. In contrast, RSV/B underwent a dramatic restructuring, with disappearance of pre-pandemic lineages and appearance of a dominant B.D.E.1 lineage in pandemic and post-pandemic periods. RSV/B exhibited higher genetic diversity and accumulated non-synonymous variants at nearly twice the rate of RSV/A, particularly in the M2-2 gene. The M2-2 gene in RSV/B showed a significant increase in non-synonymous mutations during the pandemic and post-pandemic periods, correlating with increased transcriptional activity. Mutations in antigenic site in the F protein, particularly in RSV/B, were also observed, with possible implications for immune evasion and resistance to treatments.

**Conclusion:**

The COVID-19 pandemic significantly impacted RSV evolution, leading to reduced genetic diversity during the pandemic and the emergence of novel lineages post-pandemic. RSV/B exhibited more dynamic evolutionary changes, particularly in the M2-2 gene, suggesting potential adaptive advantages. These findings highlight the importance of continued genomic surveillance to monitor the impact of emerging interventions, such as vaccines and monoclonal antibodies, on RSV evolution and public health.

**Disclosures:**

Pedro A. Piedra, MD, Gilead: Honoraria|GSK: Grant/Research Support|Icosavax: Grant/Research Support|Merck: Advisor/Consultant|Merck: Grant/Research Support|Moderna: Advisor/Consultant|Novavax: Grant/Research Support|Pfizer: Advisor/Consultant|Sanofi-Pasteur: Advisor/Consultant|Sanofi-Pasteur: Grant/Research Support|Shionogi: Advisor/Consultant|Shionogi: Grant/Research Support

